# Supplementary material for: Engineering a Bi-Conical Microchip as Vascular Stenosis Model
Source: Micromachines (Basel). 2019 Nov 18;10(11):790. doi: 10.3390/mi10110790 (PMC6915513; doi:10.3390/mi10110790)
Supplement: Supplementary file 1 [file micromachines-10-00790-s001.zip › micromachines-632252 Supplementary for conversion/micromachines-632252 Supplementary for conversion.docx]

Supplementary Materials: Engineering a Bi-Conical Microchip as Vascular Stenosis Model

Yan Li ^1,†,^*, Jianchun Wang ^1,†^, Wei Wan ^1,†^, Chengmin Chen ^1^, Xueying Wang ^2^, Pei Zhao ^1^, Yanjin Hou ^1^, Hanmei Tian ^1^, Jianmei Wang ^1^, Krishnaswamy Nandakumar ^1,3^ and Liqiu Wang ^1,4,^*

^1^ Energy Research Institute, Qilu University of Technology (Shandong Academy of Sciences), Jinan 250014, China; wangjc@sderi.cn (J.W.); wanw@sderi.cn (W.W.); chencm@sderi.cn (C.C.); zhaop@sderi.cn (P.Z.); houyj@sderi.cn (Y.H.); tianhm@sderi.cn (H.T.); wangjm@sderi.cn (J.W.); nandakumar@lsu.edu (K.N.)

^2^ Key Laboratory of Interfacial Reaction & Sensing Analysis in Universities of Shandong, School of Chemistry and Chemical Engineering, University of Jinan, Jinan 250022, China; chm_wangxy@ujn.edu.cn

^3^ Cain Department of Chemical Engineering, Louisiana State University, Baton Rouge, LA 70803, USA

^4^ Department of Mechanical Engineering, The University of Hong Kong, Hong Kong

^†^ The authors contribute equally to this work.

***** Correspondence: liyan@sderi.cn (Y.L.); lqwang@hku.hk (L.W.); Tel.: +86-531-8872-8328 (Y.L.); +852-3917-7908 (L.W.)


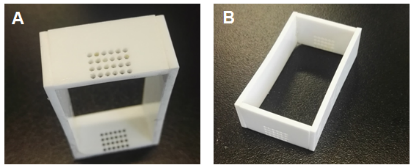


**Figure S1.** Side and top view of the plastic frame.

**Figure S2.** The relationship between tapered capillary tubes deformation ratio and the number of tapering cycles.


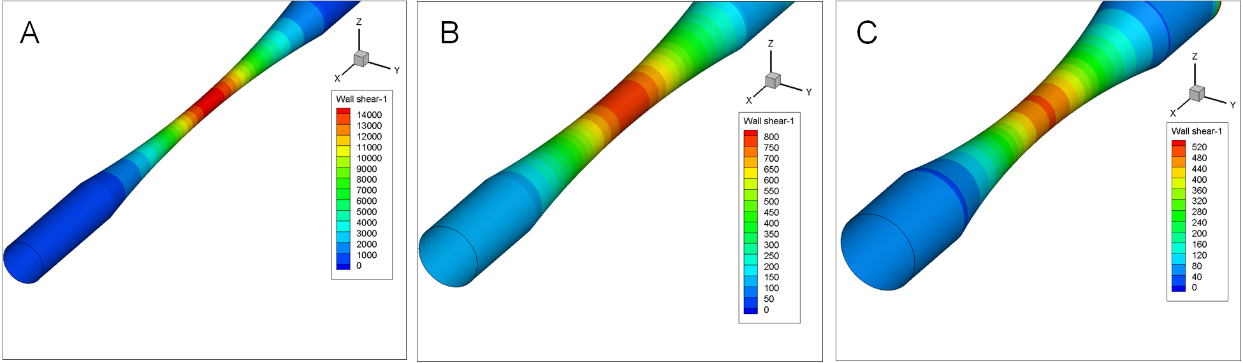


**Figure S3.** Computational simulation of wall shear rates in microfluidic channels with a stenotic shape. Computational fluid dynamics analysis displayed WSR within the channel bottom in a microchannel. (**A**–**C**) Templates inner diameters were 390 μm, 330 μm and 125 μm, respectively, and the stenosis percentage range from 67.2% to 84.5%.

**Table S1.** Simulated wall shear rates in micro-channels.

| **Case** | **Widest Diameter (μm)** | **Deformation Length (μm)** | **Narrowest Diameter (μm)** | **Percentage of Stenosis** | **Wall Shear Rates at the Narrowest Point (s^−1^)** |
| --- | --- | --- | --- | --- | --- |
| S1 | 318 | 2970 | 125 | 84.5% | 14,000 |
| S2 | 576 | 3620 | 330 | 67.2% | 800 |
| S3 | 820 | 3910 | 390 | 77.4% | 520 |
