# Supplementary material for: Engineering a Bi-Conical Microchip as Vascular Stenosis Model
Source: Micromachines (Basel). 2019 Nov 18;10(11):790. doi: 10.3390/mi10110790 (PMC6915513; doi:10.3390/mi10110790)
Supplement: Supplementary file 1 [file micromachines-10-00790-s001.zip › micromachines-632252 Supplementary for conversion/micromachines-632252 Supplementary for conversion.pdf]

micromachines

Supplementary Materials: Engineering a Bi-Conical Microchip as Vascular Stenosis Model

Yan Li [1,†,*], Jianchun Wang [1,†], Wei Wan [1,†], Chengmin Chen [1], Xueying Wang [2], Pei Zhao [1], Yanjin Hou [1], Hanmei Tian [1], Jianmei Wang [1], Krishnaswamy Nandakumar [1,3] and Liqiu Wang [1,4,*]

1. Energy Research Institute, Qilu University of Technology (Shandong Academy of Sciences), Jinan 250014, China; wangjc@sderi.cn (J.W.); wanw@sderi.cn (W.W.); chencm@sderi.cn (C.C.); zhaop@sderi.cn (P.Z.); houyj@sderi.cn (Y.H.); tianhm@sderi.cn (H.T.); wangjm@sderi.cn (J.W.); nandakumar@lsu.edu (K.N.)
2. Key Laboratory of Interfacial Reaction & Sensing Analysis in Universities of Shandong, School of Chemistry and Chemical Engineering, University of Jinan, Jinan 250022, China; chm_wangxy@ujn.edu.cn
3. Cain Department of Chemical Engineering, Louisiana State University, Baton Rouge, LA 70803, USA
4. Department of Mechanical Engineering, The University of Hong Kong, Hong Kong

† The authors contribute equally to this work.

* Correspondence: liyan@sderi.cn (Y.L.); lqwang@hku.hk (L.W.); Tel.: +86-531-8872-8328 (Y.L.); +852-3917-7908 (L.W.)

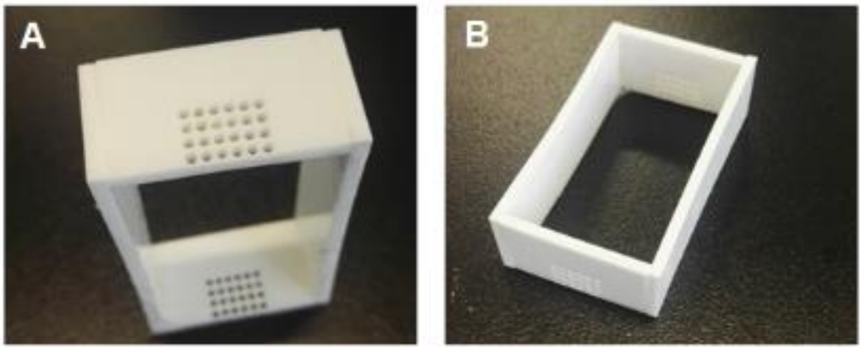

Figure S1. Side and top view of the plastic frame.

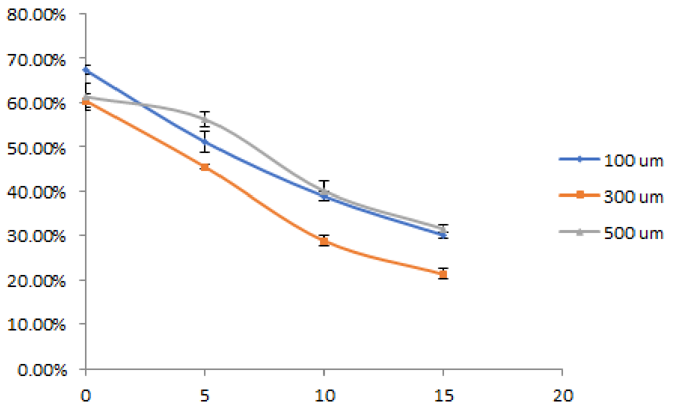

Figure S2. The relationship between tapered capillary tubes deformation ratio and the number of tapering cycles.

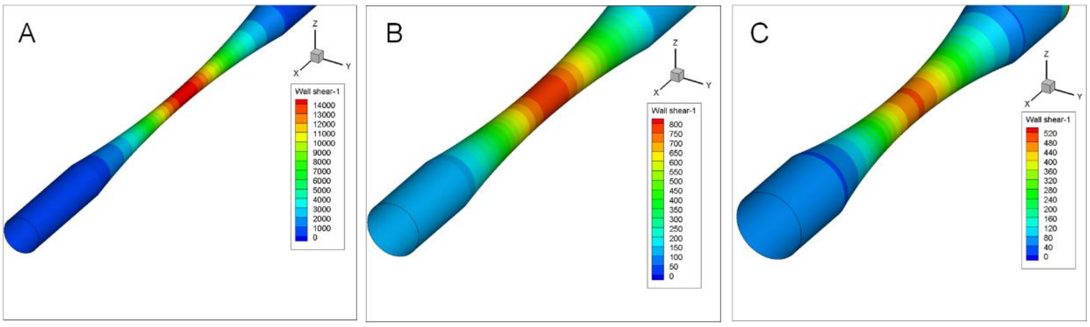

Figure S3. Computational simulation of wall shear rates in microfluidic channels with a stenotic shape. Computational fluid dynamics analysis displayed WSR within the channel bottom in a microchannel. (**A–C**) Templates inner diameters were 390 μm, 330 μm and 125 μm, respectively, and the stenosis percentage range from 67.2% to 84.5%.

Table S1. Simulated wall shear rates in micro-channels.

Case	Widest Diameter (μm)	Deformation Length (μm)	Narrowest Diameter (μm)	Percentage of Stenosis	Wall Shear Rates at the Narrowest Point (s^{-1})
S1	318	2970	125	84.5%	14,000
S2	576	3620	330	67.2%	800
S3	820	3910	390	77.4%	520
